# Case report: A systematic approach for the forensic evaluation of child abuse: lessons from a complex case

**DOI:** 10.3389/fpsyt.2026.1631508

**Published:** 2026-02-03

**Authors:** Pierpaolo Di Lorenzo, Emanuele Capasso, Anita Sammarco, Maria Pieri, Claudia Casella

**Affiliations:** 1 Department of Advanced Biomedical Sciences, University of Naples Federico II, Naples, Italy; 2 Legal Medicine Unit, ASL Roma 6, Rome, Italy

**Keywords:** child abuse, physical maltreatment, neglect, cocaine, substance abuse, guidelines, child abuse diagnosis, sodium valproate

## Abstract

Child abuse (CA) remains a significant public health concern, where distinguishing between accidental and intentional injury is particularly difficult in complex cases involving underlying medical conditions. We present a disturbing case study of severe abuse and neglect to illustrate the necessity of a systematic and evidence-based approach to forensic evaluation. The case featured a complex clinical picture: multiple bone fractures in a child with Infantile Epileptic Spasms Syndrome (West Syndrome), under treatment with valproic acid, and evidence of cocaine exposure. A systematic diagnostic work-up, structured according to authoritative international protocols, was conducted, including detailed medical history, physical examination, imaging, toxicological, and genetic analyses. Despite these compounding challenges, a careful clinical evaluation and critical review of international guidelines led to a definitive diagnosis of child abuse and neglect. This work represents a valuable educational resource for healthcare professionals and underscores the significance of implementing systematic protocols for assessing child maltreatment.

## 1 Introduction

Child abuse (CA) is a global social and health issue. An accurate estimation of its incidence is complex due to several reasons, among which terminological ones (1). In this regard, the World Health Organization defines CA as: “all forms of physical and emotional ill-treatment, sexual abuse, neglect, and commercial or other exploitation that result in actual or potential harm to the child's health, development or dignity” (2). Child maltreatment has the potential to result in notable physical and psychological morbidity. It is considered a major cause of death among children: in 2011 only in U.S. an incidence of 9.1 per 1,000 children died from child abuse and neglect, but such an incidence likely underrepresents the real one due to lack of recognition (3). Survivors may continue the cycle of violence or experience poor health into adulthood (4, 5). Notably, CA has been identified to account for 44.6% of mental disorders in childhood and 32% occurring throughout the life course (6–8).

Most European hospitals (51.9%) are insufficiently prepared to identify child maltreatment (9). Physicians frequently fail to seize opportunities at such a stage when the abuse can be identified and an intervention can be performed, especially as related to toddlers, underestimating the value of hallmark signs (10–12). Most health workers have never received formal training in investigating child abuse and reporting it to judicial authorities.

CA detection is a very challenging issue also considering childhood unintentional injuries that can commonly occur (13). Most of them are not due to abuse or neglect or are nonspecific, and thus it is difficult to distinguish between inflicted versus non-inflicted trauma.

In addition, the child victims may not always be cooperative, for example if they are preverbal or in case they are threatened about disclosure. Besides, doctors may feel intimidated by disbelieving the caregivers of the children. According to reports from numerous authors, doctors also lack sufficient knowledge on the topic, as many have stated they were not adequately trained to address the problem (14).

Given the severe legal implications for families, an accurate and extremely cautious investigation should be conducted before assessing that CA has taken place.

Despite the severe legal implications, there is currently a critical absence of systematic standardization for the diagnostic work-up in suspected Child Abuse (CA) cases. If the history provided and the nature of the injury do not correlate, the forensic pathologist must conduct a scrupulous, impartial investigation, fully documented by instrumental and laboratory data. Beyond traditional assessments, comprehensive documentation requires photography of all medical examination findings.

Systematic reviews (15) highlight the marked heterogeneity across authoritative international guidelines regarding CA management. This variability stems from inconsistent policies, differing accessibility to diagnostic tests, and a fundamental lack of consensus on key terminology, such as the definition and scope of sentinel lesions. This procedural fragmentation creates significant challenges for professionals, hindering the standardization of practice and making a definitive diagnosis difficult. The novelty of our work resides in proposing a systematic, step-by-step medico-legal assessment protocol. This protocol provides a hierarchical and replicable framework for integrating diverse data and strengthening the evidence base required for judicial authority.

This is necessary to ensure clarity for judges and non-medical professionals and to standardize the work of forensic doctors (16).

Twenty clinical guidelines published by academics and health agencies in 15 high-income countries between 2010 and 2020 on the diagnosis of CA were evaluated (17). The authors intended to carry out a systematic evaluation of the completeness of such guidelines to support evidence-based guidance into an algorithm for CA diagnosis. It was shown that their analysis might explain the variability between countries in CA clinical guidelines, both by territorial variations in epidemiological characteristics of diseases and by different accessibility to diagnostic tests.

Comparing the contents of the various guidelines, there is no uniformity among the authors on the definition of "sentinel lesion”. Some authors define it as skin lesions (18–23) such as bruises or burns: these are the most common injuries, but also the most underestimated, since physicians don’t deepen beyond the suspicious areas (identified by the “TEN rule (24)”: torso, ears, neck) losing until 19% of the diagnoses (25, 26). In the concept of the sentinel lesions, other criteria (27–34), include intraoral or abdominal traumas, intracranial lesions, and bone fractures. Some authors point to skeletal injury as the most important sign of CA (35, 36).

Additionally, there was no agreement among the different protocols on the position, size, and number of what should constitute a sentinel lesion.

Blood investigations and radiological assessments are always considered helpful in delaying a proper diagnosis, as they allow reference to objective and scientific parameters (17). Almost all guidelines suggest instrumental investigations, including bone X-rays, skull CT, head MRI, or ocular fundus examination. However, not all of them recommend performing these tests systematically (15).

Regarding laboratory tests, only some authors systematically envisage exploring the coagulation panel (18, 37–39) (although there is no conformity in the examined parameters among the different guidelines) or investigating bone metabolism (18, 40–43).

While we acknowledge the comprehensiveness of guidelines such as those from NICE (44) our application to a complex clinical scenario demonstrated the persistent need for a more structured procedural checklist to bridge the gap between broad international recommendations and rigorous forensic application.

The novelty of our work resides in proposing a systematic, step-by-step medico-legal assessment protocol. This protocol transcends the existing guidelines by providing a hierarchical and replicable framework for integrating diverse data (clinical, toxicological, radiological, and genetic) to mitigate diagnostic errors and ensure procedural consistency, thereby strengthening the evidence base required for judicial authority.

## 2 Case presentation

### 2.1 Anamnesis and circumstantial data

We report the case of an eight-year-nine-month-old girl with a diagnosis of West syndrome (currently known as infantile epileptic spasms syndrome), a form of infantile myoclonic encephalopathy. She was admitted for poor psychophysical condition.

This child was subjected to hospitalization in a national children's hospital for the diagnosis of "dehydration and poor hygienic conditions". Forensic expertise was necessary for suspected child abuse.

Firstly, the examination of the medical record was made, especially for the anamnestic data. Only a few days before hospitalization, the child had been put in a foster home because of the verified habitual drug use of her parents.

Circumstantial data showed that the child had never attended school, had never received the compulsory vaccinations obligatory in Italy for school-age children, and she did not even have a health card.

The review of her clinical history evidenced she was born by eutocic delivery and had been admitted to hospital at two months with sepsis. Anti-epileptic therapy started two months later when the child was hospitalized with an episode of psychomotor arrest. A diagnosis of infantile epileptic spasms syndrome was made, and a therapy with sodium valproate stated.

Past medical reviews also evidenced a history of multiple hospital admissions for bones fractures. She had been admitted to the hospital in July, two years before our clinical examination, for a fracture of the left distal femur, which was treated with reduction and cast immobilization. In the same year, in November, a fracture of the right femur was diagnosed and surgically treated. Both fractures were attributed to epileptic seizures. In the same year, in December, the child was hospitalized again because of the re-fracture of the left femur, which was treated by a plaster cast. A month later, a further fracture of the right femur occurred.

During the hospitalization, efforts to contact the child’s parents were unsuccessful, so it was not possible to talk with them. In addition, the poor cognitive development of the child and her difficulty in speech precluded any form of communication.

### 2.2 Physical examination and initial clinical findings

The careful clinical examination evidenced as the girl was bedridden and tube-fed, with bandaged wrists. Her hair was unkempt, and it looked as though it had been cut unskillfully with scissors; there was every appearance of asymmetrical lengths. The hygienic state was poor; she was in diapers. There was extensive dermatitis and contusion of the right arm, compatible with venipuncture.

**Figure 1:**
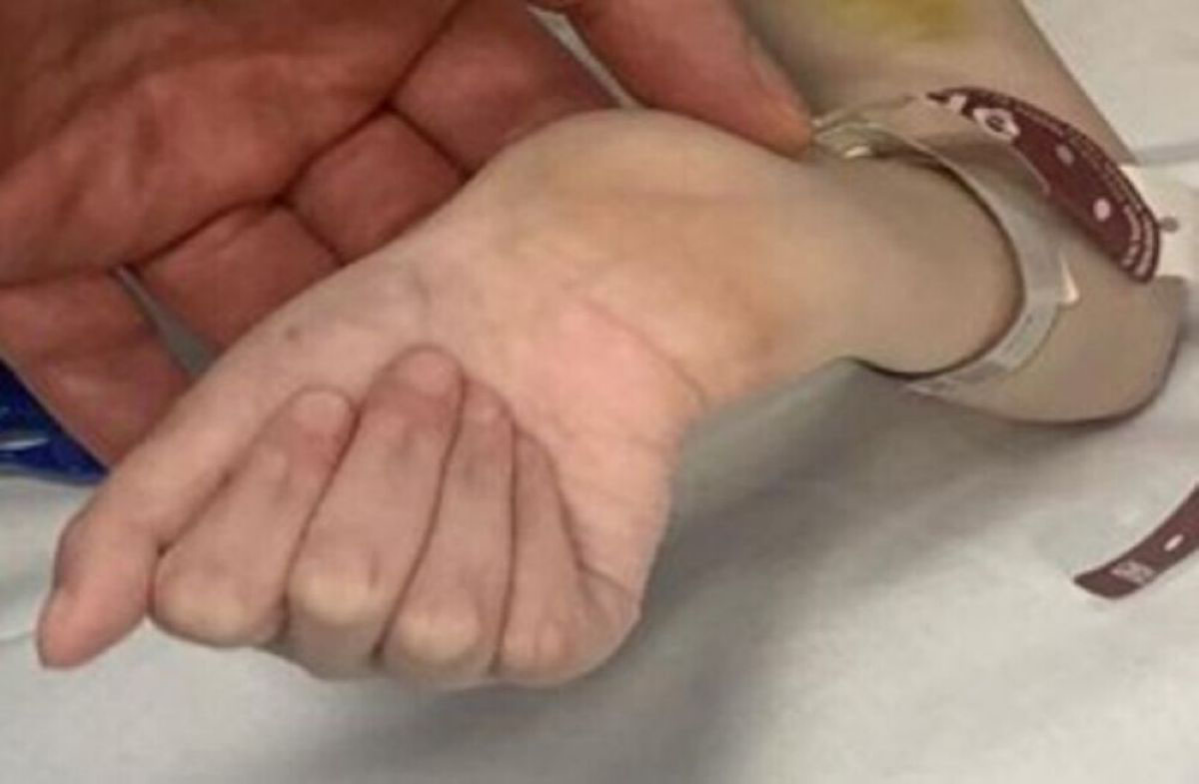
Right wrist (ulnar side of the right wrist in extension and ulnar inclination).

The child was underweight (13.7 kg) and severely malnourished. Her right wrist was dysmorphic, as in the case of chronic fracture that has been neglected and poorly treated, presenting extension, ulnar deviation, and palmar laxity (**Figure 1**).

There was also an ulnar deviation deformity of the contralateral wrist, but the presence of a bandage did not allow further evaluation.

**Figure 2:**
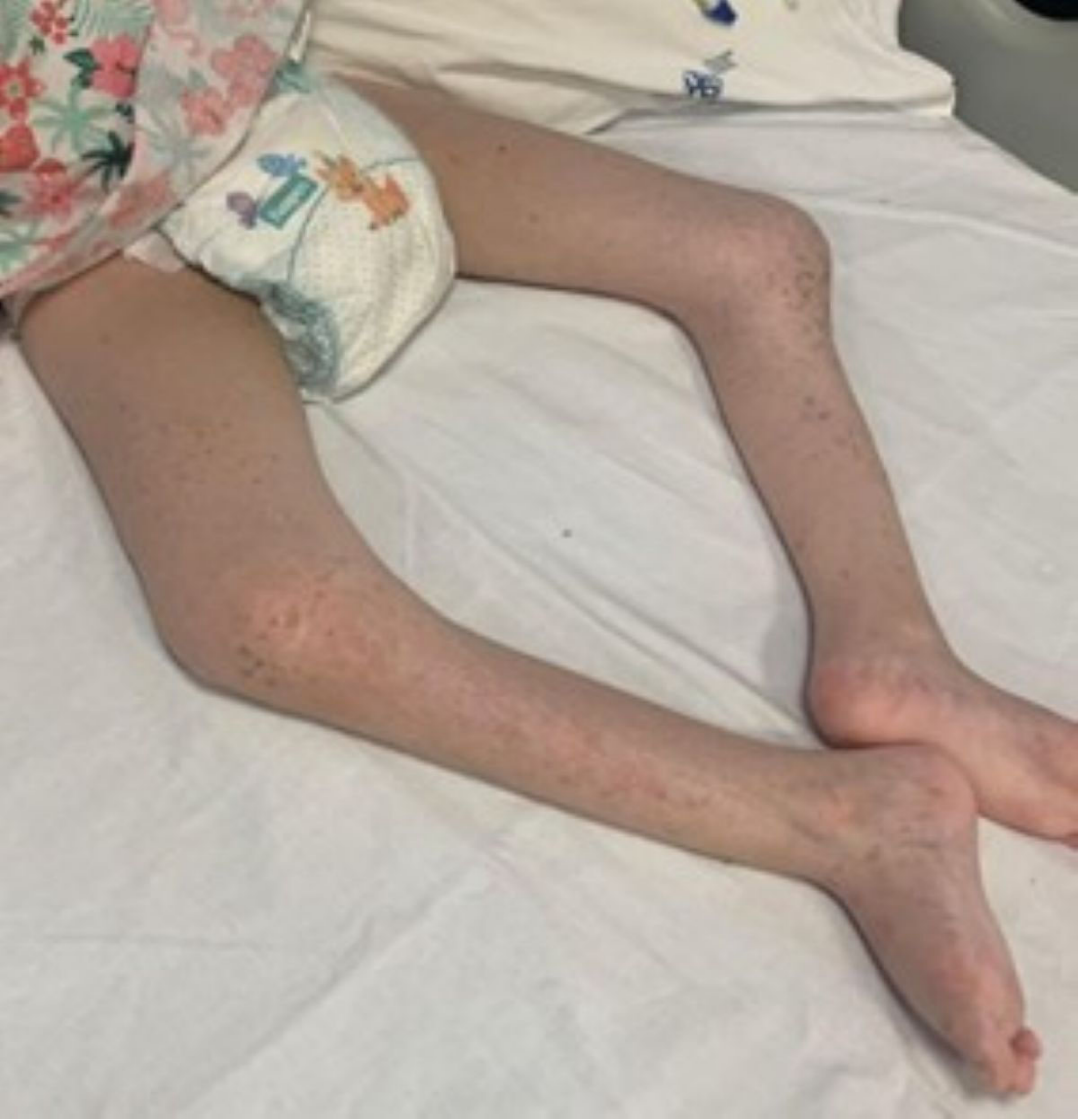
Lower body (hips and legs deformation with feet in semipronation).

Both hips were flexed and externally rotated, while the knees showed flexion and scars at the level of the lower third of the inner thigh. The legs showed an arcuate deformity, with anterior convexity in the lower third and semi-pronation of the feet (**Figure 2**).

Genital examination showed dermatitis in the gluteal and vulvar area.

### 2.3 Laboratory and radiological assessment

The hematological tests showed severe anemia (Hb 6.50 g/dL) and vitamin D deficiency (vitamin D 4.49 ng/mL), while the inflammatory markers were increased: CRP 85.09 mg/L, procalcitonin 5.86 ng/mL.

**Figure 3:**
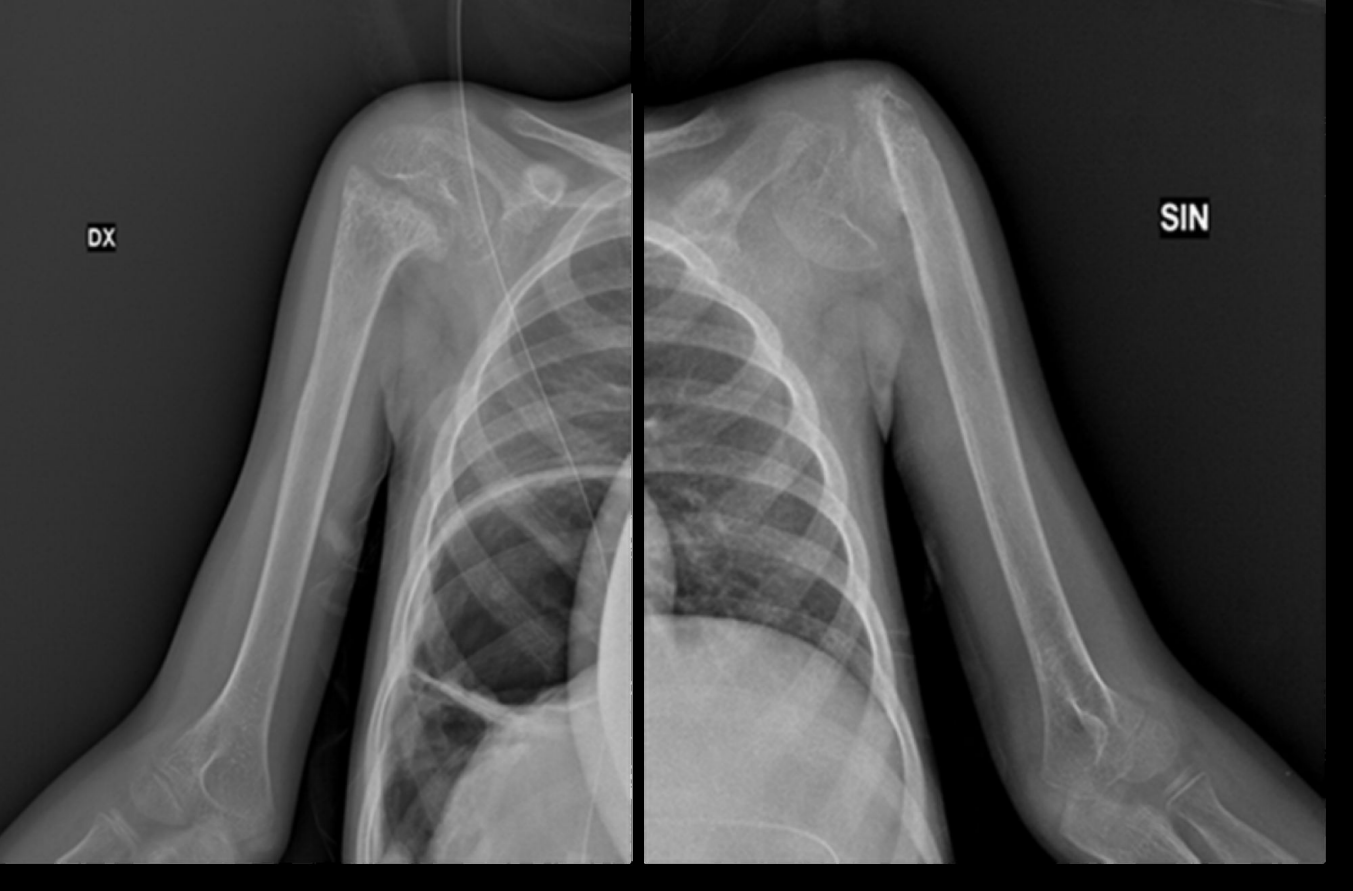
Humerus (fracture outcomes of the surgical neck fragments of the two humerus).

The X-rays performed during the hospitalization showed diffuse osteopenia. Dysmorphic appearance of the examined skeletal segments was present including fracture sequelae with angulation of the surgical neck fragments of both humeri (**Figure 3**).

**Figure 4:**
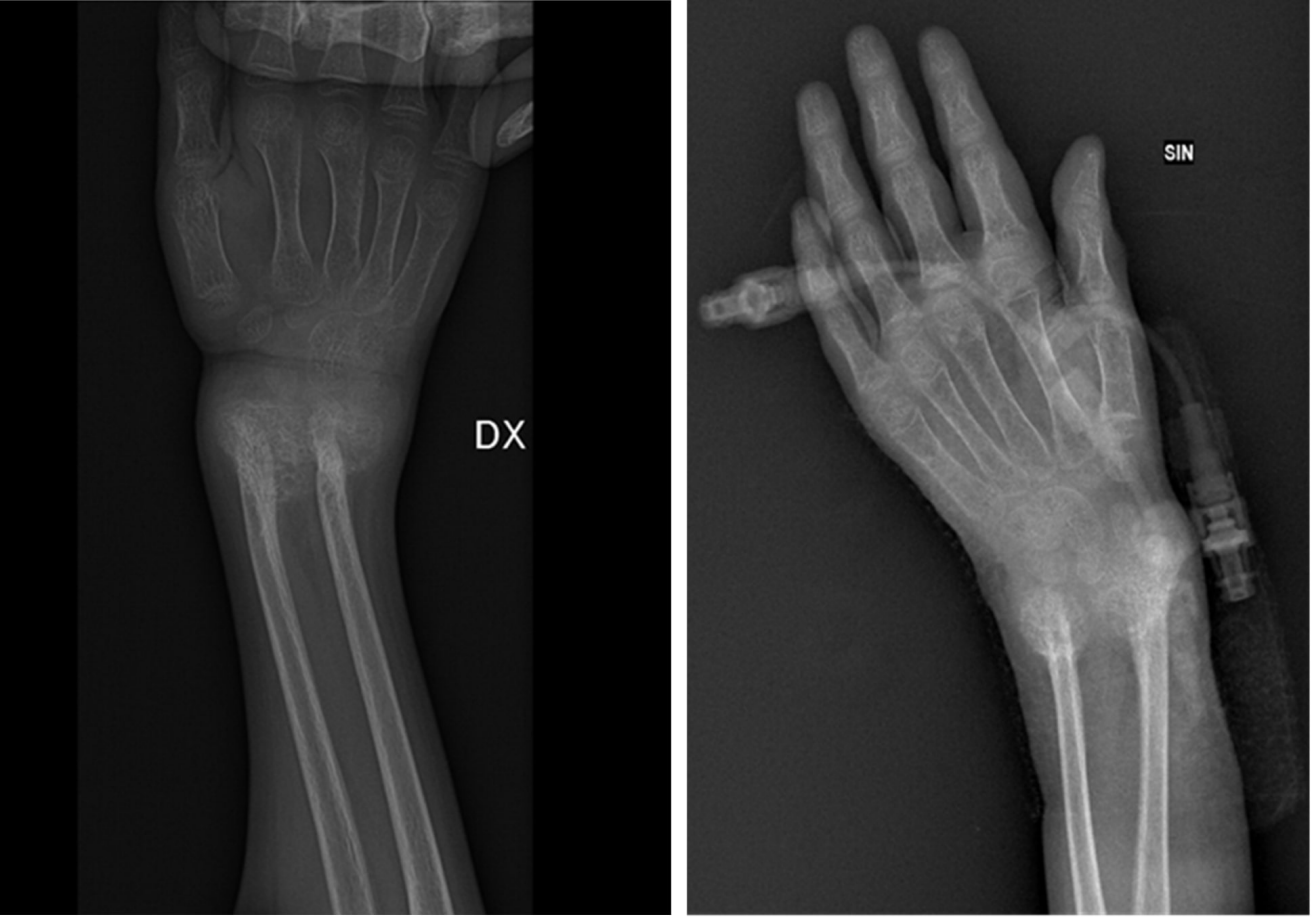
Radius and ulna (fracture outcomes of the distal metaphyses of the right and left radius and ulna).

Moreover, fracture outcomes of the distal metaphyses of the right and left radius and ulna were appreciated (**Figure 4**).

**Figure 5:**
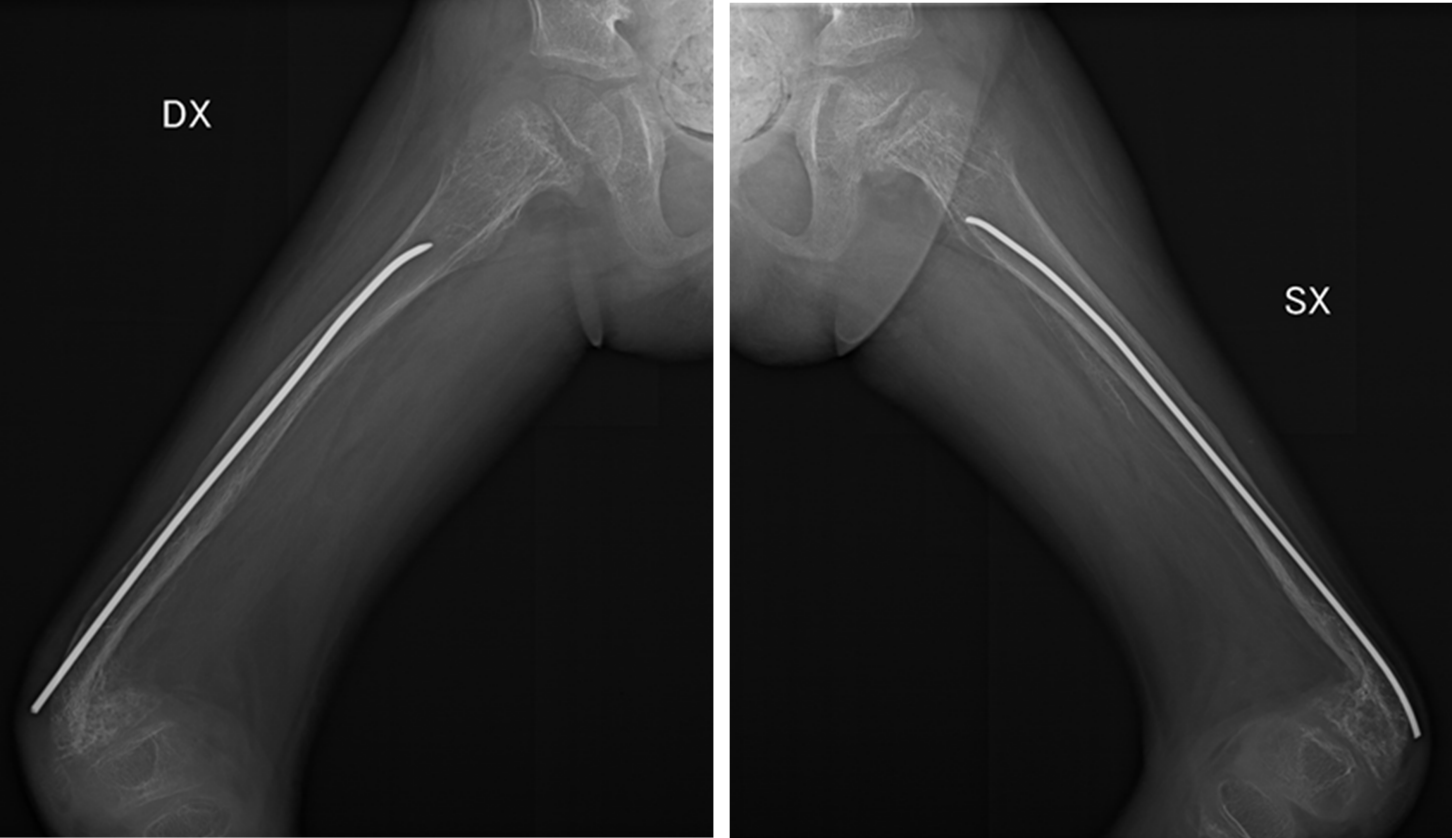
Femurs (fracture outcomes of the femurs).

The radiography also revealed bilateral femoral fracture dysmorphic outcomes (**Figure 5**).

**Figure 6:**
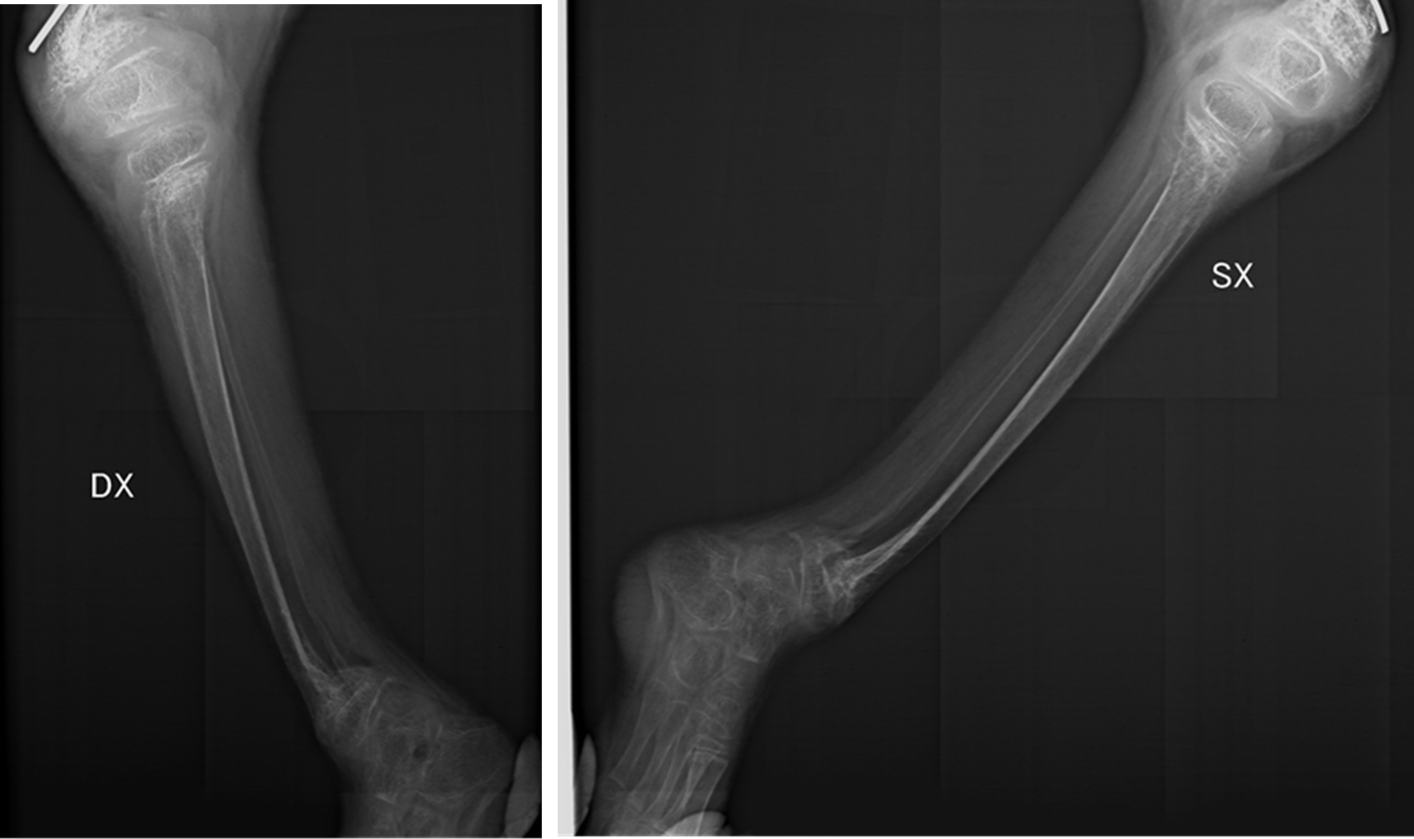
Tibia (radiographic image of right and left tibia dimorphism with angulated distal segments).

A bilateral tibia dimorphism with angulated distal segments was shown too (**Figure 6**).

All these findings were suggestive for traumatism outcomes.

Furthermore, fractures of the humerus, radius, ulna, and tibia were not assessed during previous hospitalizations. These data suggest that these fractures may have occurred within the 18 months prior to their radiographic evaluation. Other investigations were carried out, such as a fundus examination, which was within normal limits.

### 2.4 Toxicological analysis and definitive diagnosis

Based on the clinical examination and history, we also requested toxicological and genetic counseling to evaluate potential narcotic substance positivity and congenital bone fragility conditions. Hair analysis revealed cocaine and its specific metabolite (benzoylecgonine) concentrations three times higher than the *cut-off* values (0.5 ng/g and 0.05 ng/g for cocaine and benzoylecgonine, respectively), across the entire length of the sample (12 cm). Analyses were performed by dividing the entire hair segment into two parts (6 cm each), both tested positive to cocaine and benzoylecgonine.

Preliminary genetic investigations have ruled out metabolic bone disease.

We summarized the most important suggestive elements we found in **Table 1**.

**Table 1:** Anamnestic, clinical, instrumental and laboratory data collected.

Clinical elements investigated	Data obtained
Past pathological history	eutocic delivery data absence of mandatory vaccination prophylaxis sepsis at the age of two months West Syndrome diagnosis, in treatment with sodium valproate from 2014 multiple bone fractures (July 2020, left distal femur’s fracture treated by cast-immobilization; November 2020, right femur fracture surgically treated; December 2020, left femur refraction treated by cast- application; January 2021 right femur fracture, untreated)
Family history and circumstantial data	parental habitual narcotic drugs ‘use entrusted to a foster home unschooled person severe interaction deficits no health card possession
Physical examination	dehydration and poor hygienic conditions underweight (kg 13.7) poor muscle trophism bedridden irregular haircut bandaged right wrist widespread skin dermatitis, including gluteal and vulva’areas dysmorphic right wrist (in extension and ulnar inclination, with palmar laxity) ulnar deformity of the left wrist hips’ external rotation decubitus, with flexed knees bilateral legs’ arcuate deformation with anterior convexity of the lower third bilateral semi-pronation feet decubitus
Laboratory exams	anemia (Hb 6.50 g/dL) vitamin D deficiency (4.49 ng/mL) positive inflammatory markers (CRP 85.09 mg/L; Procalcitonin 5.86 ng/mL)
Radiographic investigations	osteopenia fracture outcomes with angulation of the surgical neck fragments of the two humerus outcomes of bilateral fracture of the distal metaphysis of the radius and ulna dysmorphic outcomes of bilateral femur fracture bilateral tibia dimorphism with angulated distal segments
Toxicological investigation	hair analysis (12 cm length, positive both for cocaine and benzoylecgonine)
Instrumental examination	normal ocular fundus
Genetic investigations	absence of metabolic bone diseases

## 3 Discussion

Our clinical review was systematically initiated by assessing the compatibility of the traumatic injuries against the provided history. The observed findings, including severe malnutrition, poor hygiene, and profound developmental delay, are classical hallmarks of profound neglect, providing a necessary baseline for interpreting the traumatic findings. While the child’s non-verbal status precluded direct communication the thorough physical examination revealed multiple skeletal deformities, irregular haircut, and widespread dermatitis.

Crucially, the presence of polytopic and polychronous fractures - meaning multiple fractures in different locations and various stages of healins - is a pathognomonic finding highly indicative of Non-Accidental Injury (NAI), particularly in older children who are non-ambulatory.

The differential diagnosis systematically excluded iatrogenic and congenital etiologies: the pattern of long bone fractures (diaphysis and distal metaphysis) differed from the typical valproate-induced osteopathy (lumbar spine and femoral neck) and genetic fragility was ruled out.

This systematic exclusion process, central to Clinical Forensic Medicine confirmed that violent external traction force was required to cause the observed injuries. Our interpretation rigorously formalizes this CFM approach, providing the structured reasoning required for judicial certainty.

It is good practice to try to engage in conversation with children to obtain information. While they can be preverbal and may be afraid and thus lie, it is nevertheless useful to inquire from the child how the injury occurred to explore if history is suggestive of child abuse. Sometimes children may show overt behavioral changes (45). In our case, we could not make use of this information as she was a non-verbal girl and quite uncooperative despite being above two years.

It is a good practice to disclose the general injuries of abuse by a thorough physical examination. Severe muscular hypotonia, an irregular haircut, diffuse skin dermatitis affecting even the gluteal and vulvar regions, and skeletal deformities were disclosed in the whole body examination.

A detailed description must be ensured, highlighting each topographical and morphological feature of each lesion. It is important that all the measurements are documented in detail accurately with a view to photography for photographic record.

Inspection of the scalp in a cranio-caudal direction may reveal traumatic wounds and alopecia while the inspection of the mouth may indicate dental trauma (46).

The skin may be observed to present hematomas, ecchymoses, bruises, lacerations, burns, wounds, and scars (44). Skin lesions are most frequently found on the face, chest, back, gluteal regions, genitals, and dorsum of the hands. They often reflect the morphology of the injurious agent; they suggest a traumatic aetiology when multiple and in different stages of evolution (21). Their location, size, and shape must be documented. It is relevant to perform differential diagnosis of skin diseases that may mimic child abuse, so that differential diagnosis is necessary (47). From a multidisciplinary point of view, we consider it essential to turn for consultations of a dermatologist in the most complicated cases to help to establish real character-traumatic or pathologic condition of a skin lesion.

After a check-up, a full neurological examination is always advisable to check correspondence with the child's developmental age (reflexes, sensory, gross and fine motor skills). In the here described case, the girl did not walk without support and hardly could keep an upright position.

A neurological check-up is mandatory also to exclude brain damage condition rather typical for maltreated infants (17).

It may also be useful to, very carefully, palpate the skeletal system, especially the upper and lower limbs for any acute or healing- bone callus-fractures.

Fractures are the most common finding in suspected child abuse (48). Being the most common sign of inflicted trauma, excluding soft tissue injuries, bone injury represents a kind of hallmark in suspected physical child abuse. Fractures occur in 17% of children under 2 years of age and are non-accidental; 84% of patients reported for abuse have multiple fractures (49).

First to be considered is the number of injuries. In an accident, a bone lesion is more likely to be isolated, whereas multiple-site lesions in various stages of healing are highly indicative of non-accidental injury. Of all fractures, long bone fractures account for 76% (50, 51).

In the context of childhood injuries, the distal metaphysis is the most vulnerable part of the bone to injury in an infant. Although children older than 18 months rarely present with metaphyseal fractures in the context of abuse-inflicted injury, this localization is considered diagnostic for child abuse since it constitutes the most vulnerable part of the bone in an infant. They most frequently occur in the distal femur, proximal tibia, distal tibia, and proximal humerus (52, 53), consistent with the case presented. The child was further subjected to skeletal X-rays that displayed diffuse osteopenia and sequelae of traumatic symmetrical fractures of all four limbs.

The X-rays performed on the fractures in this eight-year-nine-month-old child show polyfocal and symmetrical characteristics, suggesting a traumatic origin with recurrence of traumatic events.

We did not exclude genetic bone disease and thus also investigated that, although preliminary results have been negative.

Laboratory tests included investigation of organ function, hemostasis, and bone metabolism. Anemia and vitamin D deficiency-the cause of increased fracture risk-were detected. Vitamin D deficiency may also lead to muscle weakness (54), which presented as difficulty to stand and walk.

More importantly, her inability to walk made the explanation of the fractures based on possible accidental events less likely.

Considering the child's pharmacological history, an iatrogenic cause of the lesions needed to be excluded. The anti-epileptic medication administered to the child, sodium valproate, is known to contribute to Vitamin D deficiency and reduction in bone mineral density (55), which is a risk factor for pathological fractures. Literature reports this risk predominantly at the level of the lumbar spine and femoral neck (56); in this case, the fracture lines occurred in the long bones of all four limbs and involved the diaphysis and distal metaphysis.

We therefore concluded that spontaneous fractures could not be solely accounted for by mere intake of sodium valproate, but indeed a violent external traction force was required to cause the various bone fractures observed in the child.

A toxicological examination of biological matrices, especially hair, was conducted in view of the intake of psychotropic substances.

We have also taken into consideration the consumption of psychotropic drugs through toxicological analysis of hair samples (12 cm length), divided into two 6 cm fragments analyzed separately.

Most authors recommend hair testing in cases of unexpected blood levels of un-prescribed drugs present in the child's body, reported ingestion of one or more toxic substances, or suspicion according to circumstantial data.

Toxicological investigations could be carried out on various biological matrices (57), each with a time window, which is the span of time from ingestion during which the substance is still detectable. We chose a keratin matrix (hair) (58), which has features such as a large detection window (from months up to 1 year) and low invasive-ness. We took one strand of about 50–100 mg of hair to provide adequate statistical weight to the data and analyzed the full hair without segmenting it. The sample was positive for cocaine and its specific metabolite (benzoylecgonine) across its entire length, indicating that the child tested positive for cocaine intake or administration at least twice in the previous year.

Our protocol advances current practice by providing a necessary structured methodology for integrating diverse data. It is summarized in the Diagnostic Protocol Suggested in **Table 2**.

**Table 2:** CA diagnostic protocol suggested.

Procedure	Description
Past and present pathological history of the child	Investigate about: habits, sleep-wake, alvus, diuresis and any previous access to the emergency room or any hospital admission medical history suggestive for traumatic events systemic pathologies linked to the injuries living environment and parents' history
Circumstantial data	it’s common a noticeable delay between the accident and the required visit find elements useful for the purposes of abuse compatibility observe the child's attitude during the visit
Interview the child (if not preverbal) and then the caregivers (separately)	Evaluate if: the medical history is compatible with the type of injury reported the version reported appears unclear or implausible the parent shows inadequate level of preoccupation in relation to the severity of the injury the parents, questioned separately, give different versions the version changes each time it is retold
General physical exam of the child	evaluate nutrition, hydration, neglect signs (hair, hygiene, teeth) search for possible signs of recent traumatic action (with particular attention to skin, skeletal and neurological injuries)
Laboratory inverstigations	complete blood count electrolytes liver and kidney function coagulation test bone metabolism
Toxicological investigation	To do where there is one or more of this elements: clinical signs of poisoning unexpected blood level of the drug family history of drug ingestion suspicious circumstantial data
Instrumental examination	fundus examination (in suspicion of shaken baby syndrome) total body skeletal x-ray dental x-ray (if we find dental lesions) neuroradiology (CT and MRI)
Genetic investigations	Perform it to exclude genetic predispositions to vasculopathies, coagulation abnormalities and bone fragility

In the first instance, according to our clinical-based approach, once the vital parameters have been recorded and the subject is stable, the visit can start with a study of the past and present pathological history, as well as the circumstantial data.

It is a good practice also proceeding to an interview first with the child and then with the caregivers, separately if there are more than one, to notice if there is any inconsistency in the narrative of the event.

A full-body physical examination is to be carried out, in which all parts of the body are examined, including the oral cavity. All suspicious findings are to be photographed and documented along with measurements in exact detail. Injuries should be clearly and in detail described in the report by the medical examiner.

For lesions of uncertain significance, such as dermatological findings, we recommend consulting a specialist.

Special attention should be given to the osteoarticular system, since this is the most common site of injury.

The neurological tests will complete the physical examination.

Finally, lab tests to assess the patient's overall health status should be ordered. Specifically, a coagulation panel, especially when bruising or internal bleeding is present, in order to identify traumatic versus spontaneous bleeding. In the presence of fractures, to rule out non-traumatic etiology, tests regarding bone metabolism are also necessary.

We recommend toxicological investigation on biological matrices. In addition, genetic investigations must be performed to exclude congenital pathologies that might exclude traumatic aetiology of the lesion.

Regarding instrumental examinations, a fundus examination should be per-formed, especially in children younger than one year of age, to rule out retinal hemorrhages associated with abusive head trauma (59).

While some authors advocate for a more conservative approach to avoid unnecessary radiation exposure in the absence of clear clinical suspicion, we believe that a skeletal X-ray is essential, even when the clinical examination appears unremarkable. The presence of polychronous and polytopic lesions, or chronic findings as seen in our case, is considered pathognomonic. In certain circumstances, a brain MRI may also be valuable.

Once a diagnosis or strong suspicion of child abuse has been made, the Judicial Authority must be informed.

## 4 Conclusions

This case serves as a critical illustration of systemic failure in the identification of CA. It is highly plausible that previous healthcare providers were negligent, as evidence of abuse and chronic neglect was systematically overlooked in prior admissions.

This highlights the need for a comparative analysis of diagnostic errors and the necessity of mandatory, standardized protocols to mitigate the risks associated with inadequate training. Furthermore, the findings carry significant forensic psychiatric implications.

The documented chronic cocaine exposure, coupled with parental substance abuse, underscores the critical link between severe substance use disorders, profound neglect, and the perpetration of physical harm.

Healthcare providers should perform a thorough check for any signs of child abuse. Documentation in the forms of writings and photographs plays an essential role in establishing the causal relationship between the alleged incident and the inflicted injuries and consequences. These are very important for both immediate child protection and diagnosis.

Once a diagnosis or strong suspicion of child abuse has been made, the Judicial Authority must be alerted immediately.

Following our assessment, the child has been transferred to a community for children managed by social services and removed permanently from the foster family.

The comprehensive investigation emphasizes the mandatory reporting obligations and potential liability of healthcare professionals in CA cases.

Our findings underscore the urgent need for interdisciplinary vigilance across medical, legal, and social sectors. The forensic pathologist occupies a singular and crucial position, required not only to conduct complete investigations to establish the diagnosis but also to assist the judicial authority in the prosecution of justice.

## Data Availability

The original contributions presented in the study are included in the article/supplementary material. Further inquiries can be directed to the corresponding author.
